# Inositol Treatment for PCOS Should Be Science-Based and Not Arbitrary

**DOI:** 10.1155/2020/6461254

**Published:** 2020-03-27

**Authors:** Scott Roseff, Marta Montenegro

**Affiliations:** ^1^South Florida Institute for Reproductive Medicine, Boca Raton, FL 33428, USA; ^2^South Florida Institute for Reproductive Medicine, Miami, FL 33143, USA

## Abstract

The aim of this paper is to critically analyze the composition of many inositol-based products currently used to treat Polycystic Ovary Syndrome (PCOS). Several different combinations of myo-inositol and D-chiro-inositol, with and without additional compounds such as micro- and macroelements, vitamins, and alpha-lipoic acid, have been formulated over the years. Such therapeutic proposals do not take various features of inositol stereoisomers into consideration. As an example, it is important to know that D-chiro-inositol treatment may be beneficial when administered in low doses, yet the progressive increase of its dosage results in the loss of its advantageous effects on the reproductive performance of women and a deterioration in the quality of blastocysts created via in vitro fertilization (IVF). In addition, we have to consider that the intestinal absorption of myo-inositol is reduced by the simultaneous administration of D-chiro-inositol since the two stereoisomers compete with each other for the same transporter that has similar affinity for each of them. A decrease in myo-inositol absorption is also found when it is coadministered with inhibitors of sugar intestinal absorption and/or types of sugars such as sorbitol, maltodextrin, and sucralose. The combination of these may require higher amounts of myo-inositol in order to reach a therapeutic dosage compared to inositol administration alone, a particularly important fact when physicians strive to obtain a specific plasma level of the stereoisomer. Finally, we must point out that D-chiro-inositol was found to be an aromatase inhibitor which increases androgens and may have harmful consequences for women. Therefore, the inositol supplements used in PCOS treatment must be carefully defined. Clinical evidence has demonstrated that the 40 : 1 ratio between myo-inositol and D-chiro-inositol is the optimal combination to restore ovulation in PCOS women. Therefore, it is quite surprising to find that inositol-based treatments for PCOS seem to be randomly chosen and are often combined with useless or even counterproductive molecules, all of which can weaken myo-inositol's efficacy. Such treatments clearly lack therapeutic rationale.

## 1. Introduction

This review aims to evaluate the composition of many inositol-based products currently used for treating Polycystic Ovary Syndrome (PCOS). Those product compositions were examined in light of the scientific evidence thus far available, and we focused our analysis on the therapeutic rationale for utilizing such compounds.

A careful MEDLINE search was conducted to identify the most significant studies on inositols used to treat women with PCOS. Furthermore, an examination of the dietary supplement market was targeted towards identifying the different products containing myo-inositol (MI) and D-chiro-inositol (DCI) alone vs. MI plus DCI along with other significant molecules used in said PCOS patients.

Important organs such as the brain need high MI concentrations (10- to 15-fold the values detected in peripheral blood) [1]. Also, the ovary uses high levels of MI to efficiently carry out its physiological activities [2].

MI can be transformed into DCI by a specific NAD/NADH-dependent epimerase, which is unidirectional and is stimulated by insulin [3, 4]. Endogenous production of both inositol isomers varies depending on the needs of the specific target tissue [5]; as an example, in normal women, the plasma ratio of MI to DCI is 40 : 1 [6], while in ovarian follicular fluid the ratio is close to 100 : 1 [7].

## 2. Inositols and the Therapeutic Target of PCOS

The research world demands a rationale for the necessary justification to carry out any scientific study, and the therapeutic rationale underlying the use of inositols in PCOS derives from their activities as insulin sensitizing molecules and their beneficial effects on metabolism [5, 8–10]. We highlight herein the two specific inositol stereoisomers, MI and DCI, as they both function as insulin second messengers and mediate different actions of insulin. MI is converted to an inositolphosphoglycan (IPG) insulin second messenger (MI-IPG) involved in cellular glucose uptake, whereas DCI is converted to an IPG insulin second messenger (DCI-IPG) involved in glycogen synthesis [11]. At the ovarian level, however, it has been shown that an MI-based second messenger is involved in both glucose uptake and FSH signaling, whereas a DCI-based second messenger is devoted to insulin-mediated androgen production. Previous studies performed by Cheang and his team [12] provided evidence that the impairment in insulin signaling in PCOS could be the result of a defect in the IPG insulin second messenger pathway, consistent with the insulinomimetic role of IPGs in activating enzymes that control glucose metabolism. In women with PCOS, a deficiency of IPGs in tissues, or altered metabolism of inositols to IPG mediators, could play a role in inducing insulin resistance [13]. The first controlled clinical trial of inositols in PCOS was published in 1999. In that study, 1200 mg of DCI vs. placebo, given orally once a day for 6–8 weeks to 44 obese PCOS women, improved insulin sensitivity and decreased circulating free testosterone levels, whereas there was no effect of placebo. DCI administration also resulted in ovulation in 19 of 22 women (86%), whereas only 6 of 22 women (27%) ovulated in the placebo group [14]. In 1998, before the study publication, Insmed Pharmaceuticals had obtained a US patent claiming the effectiveness of DCI in the treatment of PCOS and, in 2002, a follow-up study was performed by the same group in lean women with PCOS [15]. Again, and in agreement with the earlier study [14], the administration of DCI was associated with improved insulin sensitivity, a reduction in circulating free testosterone, and increased frequency of ovulation [15]. Insmed Pharmaceuticals subsequently embarked on a large multicenter placebo-controlled trial of DCI in women with PCOS using a dose of DCI twice as high as ever previously used (i.e., 2400 mg).

However, the results were never published and were very surprising and disappointing. The higher dose of DCI failed to reproduce the outcomes of the two previous studies [14, 15] in terms of improving ovulatory frequency. The lack of efficacy in the latter trial was attributed to the higher dose of DCI administered. Consequently, the company gave up proceeding with the use of DCI in clinical trials on PCOS.

Previous studies had highlighted the pivotal role of MI administration for enhancing the success of in vitro human fertilization (IVF) [16]. It was also reported that follicular fluid (FF) volume and its content of MI were significantly higher in follicles containing mature and subsequently fertilized oocytes compared with follicles with immature and oocytes retrieved but unfertilized. Furthermore, the levels of MI in FF were positively correlated with embryo quality [17].

In 2007, a randomized controlled trial (RCT) was carried out with MI in 30 PCOS women undergoing ICSI; patients were administered 4 g MI daily starting from the day of Gonadotropin Releasing Hormone (GnRH) administration. Treated patients, compared with controls, obtained an increased frequency of spontaneous menstrual cycles, and this finding suggested that MI may be useful in the treatment of infertility in PCOS [18]. Several follow-up studies supported these findings and the idea that MI exerts beneficial effects on ovulation and oocyte quality [19–22]. Of note, MI administration to women with PCOS undergoing IVF was associated with a reduction in the total quantity of recombinant FSH (rFSH) administered and the number of days of stimulation [23]. These evidences demonstrate that MI improves FSH sensitivity, lending further support to the idea that MI administration beneficially affects ovarian function and oocyte development. Unfer and coworkers conducted a comparative study of the effects of administration of MI versus DCI on oocyte quality in PCOS patients. They reported that the number of mature oocytes was significantly higher, with a parallel diminution in the number of immature oocytes, in the MI group compared to the DCI group, even though the total number of oocytes retrieved did not differ between the two treatment groups [24]. A potential explanation for this phenomenon is the tissue-specific nature of insulin resistance in women with PCOS. Indeed, although muscle and liver are insulin resistant in women with PCOS, the ovaries retain normal insulin sensitivity, highlighting the tissue-specificity of insulin resistance in PCOS. This is the so-called “DCI paradox” in the ovary [25], proposed by Unfer and coworkers. In fact, the epimerase enzyme converts MI to DCI in the ovary, and ovarian epimerase is stimulated by insulin. Unfer et al. suggested that, in women with PCOS, hyperinsulinemia likely stimulates epimerase activity in the ovary, resulting in an overproduction of DCI and a concomitant depletion of MI. The authors postulated that the resulting deficiency of MI could be responsible for the poor oocyte quality and the impairment of the FSH signaling. Clearly, DCI supplementation would be ineffective (if not harmful) in such women as they already have high levels of this molecule in the ovary.

## 3. A Long Clinical Experience of MI/DCI Ratio in the Treatment of PCOS

During the past several years, starting from the finding that the plasma MI/DCI ratio in normal women is approximately 40 : 1, several clinical studies tested this ratio and found that it obtains the best effect to induce ovulation in PCOS patients. The authors found that although MI allows us to achieve satisfactory results, the 40 : 1 ratio improves this performance. It was suggested that the cooperation between the two stereoisomers can induce two important effects: (1) a DCI-mediated improvement of insulin sensitivity in liver and muscle with a consequent reduction in circulating insulin and (2) the reestablishment of MI levels in the ovary, resulting in the restoration of FSH sensitivity and in a better oocyte quality.

A recent meta-analysis [26] evaluated the efficacy of treatments with MI, alone or combined with DCI (40 : 1 ratio of MI : DCI) for 12–24 weeks, in nine RCTs comprising 247 cases and 249 controls [19, 20, 22, 27–32]. The authors took into consideration fasting insulin concentrations as a primary outcome, with HOMA index, testosterone, androstenedione, and sex hormone-binding globulin (SHBG) plasma levels as secondary. Significant reductions in fasting insulin (standardized mean difference = −1.021 *μ*U/mL, 95% CI: −1.791 to −0.251, *P*=0.009) and HOMA index (standardized mean difference = −0.585, 95% CI: −1.145 to −0.025, *P*=0.041) were found after inositol supplementation. The meta-analysis clearly demonstrated the efficacy of the therapy. In particular, a slight trend toward testosterone decrease was observed with respect to controls, whereas androstenedione levels remained unchanged. Finally, MI was able to significantly increase SHBG levels only after at least 24 weeks of administration (standardized mean difference = 0.425 nmol/L, 95% CI: 0.050–0.801, *P*=0.026). This evidence strongly suggests that the findings on the primary outcome are conclusive. Concerning the androgenic hormones, the different effects obtained on androstenedione and testosterone levels should be investigated more in depth by dedicated studies. The authors recommended avoiding exclusive DCI supplementation for three reasons: (a) high doses of DCI/day are detrimental for ovaries and oocyte maturation (see additional data below); (b) epimerase works in a unidirectional fashion; consequently, DCI cannot be reconverted into MI and therefore the action of the latter is lost; (c) MI and MI-IPG deficiencies are correlated with many insulin resistance conditions. In conclusion, the meta-analysis gave new and strong support to the supplementation of MI to improve the metabolic profile of PCOS patients.

Another systematic review and meta-analysis [33] confirmed the efficacy of myo-inositol alone or in combination with DCI in PCOS women. Those authors highlighted that various studies demonstrated the role of DCI at low dosage to increase insulin sensitivity and ovulation regularity and to reduce the levels of lipid biomarkers and serum androgen.

## 4. Latest Studies

Some recent preclinical and clinical studies have allowed a more complete picture concerning the different efficacies of the various ratios between MI and DCI in PCOS women.

A preclinical study [34] was recently carried out in a PCOS animal model. Female mice, subjected to continuous light for 10 weeks, developed an androgenic‐like phenotype of their ovaries as we find in PCOS women. The study provided the first experimental evidence that the efficacy exerted by various MI/DCI ratios (5 : 1, 20 : 1, 40 : 1, and 80 : 1) changes, supporting the metabolic link between the two stereoisomers, specifically for PCOS. The daily treatment of mice with 420 mg/kg MI/DCI in a 40 : 1 molar ratio allowed investigators to obtain a rapid and almost full recovery from PCOS signs and symptoms. Since theca cell layer hypertrophy is a hallmark of PCOS and is strongly associated with an increased production of androgens [35], it is noteworthy that the ovaries from treated mice recovered normal histological features with a reduced ratio between theca and granulosa cell layer thickness (TGR). This means that the androgenic phenotype was efficaciously reversed. The other MI/DCI ratios were less effective or even exerted negative effects on the clinical pathological conditions (obviously, the total amount of inositols administered was the same). In particular, the formulation with high DCI content demonstrated to be unfavorable with worsening PCOS features.

Moreover, a 2019 published clinical trial [36] was performed to directly compare the efficacy of seven different ratios between MI and DCI in PCOS therapy. Fifty-six patients (8 for each group) were treated orally using the following formulations: DCI alone and in 1 : 3.5, 2.5 : 1, 5 : 1, 20 : 1, 40 : 1, and 80 : 1 MI/DCI ratios. They received 2 g of inositols twice a day for 3 months. The primary outcome was ovulation, and the secondary outcomes included the improvement of FSH, LH, sex hormone-binding globulin (SHBG), 17-beta-estradiol (E2), free testosterone, and basal and postprandial insulin levels, as well as HOMA index, BMI, and menses. The authors found that the 40 : 1 MI/DCI ratio was the best for PCOS therapy directed at restoring ovulation and normalizing important parameters (progesterone, LH, SHBG, estradiol, and testosterone) in those patients. These results were significant. The other formulations were less effective. In particular, a decreased activity was observed when the 40 : 1 ratio was modified in favor of DCI. Clearly, these findings completely agree with the preclinical study of PCOS mice.

## 5. Two Explanations

The concerning decrease in efficacy found in PCOS treatment when patients were administered high doses of DCI may be explained by examining some biological mechanisms. Here, we discuss two key points for the reader, both useful in understanding this phenomenon. The first one concerns the biological local effect of DCI on blastocyst quality, while the second is related to the pharmacokinetics of MI administered alone vs. being with DCI vs. being with other substances.

### 5.1. Blastocyst Quality

A published study successfully related the concentrations of MI and DCI in follicular fluid (FF) to the quality of blastocysts. That was the first time that the concentrations of MI and DCI in FF were directly correlated with blastocyst quality. It demonstrated that DCI concentrations above the MI/DCI limit ratio of 70 : 1 in follicular fluid decreased blastocyst quality [37]. The oocytes were taken from strictly healthy young women (egg donors) who were subjected to ovarian stimulation, whereas the sperm used for fertilization was given by the male partner of each couple undergoing IVF (all men were normospermic). Good quality blastocysts, classified by grades 4 and 3, were correlated with higher percentages of MI/DCI found in FF and with more satisfactory results in IVF compared to blastocysts assessed to be of poor quality, classified by grades 2 and 1. FF specimens were allocated into two groups correlated with grades 4 + 3 and grades 2 + 1 blastocysts, respectively. Data analysis determined that the good quality threshold related to the ratio between MI and DCI in FF is basically very close to or higher than 70 : 1 (and up to 100 : 1). The reduction of the ratio under this value was shown to exert negative consequences on blastocyst quality, assessed as grades 2 and 1 blastocysts. In conclusion, a higher MI/DCI ratio in FF correlated positively with good quality blastocysts, therefore constituting a promising parameter for the success of embryo implantation and pregnancy in IVF patients undergoing intracytoplasmic sperm injection (ICSI).

This event is explainable since the DCI-IPG second messenger serves as the signal transduction system for the stimulation—due to insulin—of human testosterone biosynthesis in theca cells [38]. This condition agrees with the higher amounts of testosterone that may be found in PCOS women in comparison with healthy subjects. As pointed out by Harwood and coworkers [39], in contrast to healthy women in whom androgens are produced equally from both the adrenal glands and the ovaries [40], in women with PCOS the ovaries are usually the major source of androgens [41]. Increased androgen levels decrease the liver production of sex hormone-binding globulin (SHBG) [42], the major circulating protein that binds testosterone, thus increasing free (biologically active) testosterone levels. These hormonal abnormalities might be related in part to obesity [43]. Recent evidence regarding DCI effects on aromatase is of great importance and adds a new piece to the mosaic of our knowledge. Sacchi and coworkers [44] stimulated primary cultures of human granulosa cells (hGCs) by insulin and tested the aromatase CYP19A1 gene expression by Real-Time PCR to find out whether DCI, after 24 h incubation, inhibited the insulin effect. They used four concentrations of DCI, namely, 1, 5, 10, and 20 nM: the first one was totally ineffective, 5 nM exerted a barely visible inhibitory activity, whereas 10 and 20 nM achieved a significant inhibitory effect that became greater with increasing DCI concentrations (slightly more than 50% reduction with 20 nM DCI). Therefore, DCI seems to decrease aromatase gene (CYP19A1) expression in a dose-dependent manner [44]. Aromatase is an enzyme involved in the transformation of androgens to estrogens; hence the inhibition of its activity ends up causing an increase in the levels of testosterone and other androgens. These observations induce one to think that DCI excess in the ovary stimulates ovarian androgen production and can help to explain the worsening of oocyte and blastocyst quality observed with high DCI levels [37].

### 5.2. MI and DCI Pharmacokinetics

A very recent study [45] evaluated the changes in the PK profile of MI absorption in humans when administered combined with DCI or with glucose transporter inhibitors, as compared to MI alone. Fasting 18 healthy volunteers received a single oral dose of MI (6 g); after seven days, the same subjects were administered MI (6 g) and DCI (1 g) together, and they were treated again after one week with MI (6 g) plus 25.5 mg phlorizin, 1.7 mg quercetin, 46.7 mg chlorogenic acid, 1193.1 mg sorbitol, 260.7 mg maltodextrin, and 42.7 mg sucralose. This study demonstrated that the absorption of MI decreases when administered with DCI, showing a reduction of the AUC 0–540 of 19.1% and a reduction of 22.3% of the *C*_max_. Although the absorption of MI can occur by a diffusion process at high MI concentrations, the uptake of inositols by cells is primarily carried out by a complex system of transporters (SMIT1, SMIT2, and HMIT) which mediate an active transport of inositols [46]. These MI transporters have different tissue distributions within the human body. So far only SMIT2 was identified in the small intestine [47]; therefore it is reputed to be the only one involved in MI intestinal absorption. Based on the data analysis of their study, the authors speculated about an inhibitory effect of DCI on MI absorption in humans. SMIT2 transports MI with an average *K*_m_ of 120–150 *μ*M (spanning between 67 and 283 *μ*M), which is consistent with MI human plasma concentration having a mean value of 32.5 ± 1.5 *μ*M, with a range of 26.8–43.0 *μ*M [48]. Conversely, although DCI is transported with an average *K*_m_ of 110–130 *μ*M (similar to that of MI), the average plasma level of DCI is less than 100 nM [46]. Therefore, DCI transport usually represents a minor physiological activity of SMIT2 due to the low concentration of DCI as compared to MI. Nevertheless, when DCI is administered at a high dosage and achieves higher concentrations it is able to compete with MI. This mechanism may explain the reason why administration of DCI in high dosage seems to be able to interfere with and inhibit the intestinal transport of MI as reported in this study [45], and this fact should be carefully considered when it becomes necessary to achieve the correct dietary supplementation of inositols.

An even greater effect in decreasing MI intestinal absorption was found when the healthy volunteers took MI plus phlorizin with the other compounds. The likely main effect was due to phlorizin, an inhibitor of glucose transport that acts as a nontransported competitive inhibitor of sodium-coupled sugar cotransporters. For this reason, it is used in the treatment of diabetes and obesity because of its induction of renal glycosuria and the blockage of intestinal glucose absorption [49]. Phlorizin provides hypoglycemic activity in humans and improves glucose metabolism. Like SGLT1, the glucose transporter of the small intestine, SMIT2, is sensitive to phlorizin, which acts as a potent inhibitor at an average Ki of 15 ± 6 *μ*M [46]. These data may explain in part the decrease of MI absorption (AUC 0–540 is 31.8% less and *C*_max_ 41.1%). In addition, we can conjecture that the additional presence of nonnegligible amounts of sorbitol, maltodextrin, and sucralose acts as inhibitors, albeit weak, for the passage through SMIT2 (which is much more specific for inositols). In this way, the reduction of MI absorption due to phlorizin is further increased, resulting in an even lower plasma concentration in contrast to the administration of MI alone. These findings provide important information for PCOS therapy and are a warning to avoid the concomitant administration of MI with sugar absorption inhibitors and with sugars, since all of these compounds negatively alter MI pharmacokinetics in the human body.

At the end of this paragraph, we would like to briefly mention two studies with alpha-lactalbumin and MI. This formulation was tested since alpha-lactalbumin increases the intestinal absorption of molecules such as MI. In addition, it exerts an anti-inflammatory activity that is beneficial in PCOS. The first study demonstrated, both in vivo and in vitro, that alpha-lactalbumin significantly improves MI intestinal absorption and bioavailability [50]. In the second study, PCOS women, who were nonresponsive (inositol-resistant patients) to MI alone, were given 2 g MI plus 50 mg alpha-lactalbumin, twice a day for three months [51]. Ovulation was the primary outcome, whereas some important laboratory parameters were secondary outcomes. At the end of the treatment, 86% patients ovulated, showing an increase of plasmatic MI levels and a significant improvement of total cholesterol, triglycerides, testosterone, free testosterone, dehydroepiandrosterone sulfate, and sex hormone-binding globulin (SHBG). Also, androstenedione decreased, although it was nonsignificant. Therefore, these studies provide meaningful results to sustain the use of alpha-lactalbumin in combination with MI.

## 6. The Situation of the Current Inositol Market for PCOS

Given this overview of the scientific evidence thus far available regarding inositol pharmacokinetics and pharmacodynamics, we were astonished when we analyzed the current inositol market for the treatment of PCOS as we find a high number of combinations between MI and DCI, often in combination with other molecules (Tables 1 and 2). In most of these cases, no therapeutic rationale or therapeutic target seems to have been taken into consideration. Aside from the 40 : 1 ratio, the other ratios are very imaginative and seem to have been randomly chosen. They are without scientific and therapeutic bases. Furthermore, the ratios with high concentrations of DCI strongly conflict with all of the unfavorable data demonstrated from the use of high therapeutic doses of DCI, and these data are well known. As previously shown, the 40 : 1 combination is well supported by many preclinical and clinical studies, while preclinical trials for the other ratios and combinations are either lacking or completely absent.

## 7. Comments on Studies with Other Ratios and Added Compounds

The other MI : DCI ratios analyzed in some other clinical studies were 3.6 : 1, 5 : 1, and 10 : 1. Mendoza carried out two studies with the 3.6 : 1 ratio [52, 53] and Brusco studied only one at 5 : 1 [54], while Januszewski looked at 10 : 1 [55]. All of these ratios were without scientific justification and were derived from an outdated vision in connection with the initial use of DCI in PCOS (and later abandoned). The results cannot be properly evaluated as the number of patients was low and, above all, there has been no further evidence to support them, while the 40 : 1 ratio was confirmed by numerous studies and researches.

Concerning the “other substances” added to MI and DCI (or only to MI), few deserve to be taken into consideration since they mostly exert too generic activity and are not specifically focused on treating PCOS. In addition, the antioxidant activities of some added ingredients exert a weak downstream effect on the therapeutic target. Therefore, the addition of macro- and microelements, several vitamins, and other molecules, despite being possibly useful in some cases, basically appears to be a pure marketing maneuver and could even impair inositol absorption.

In our discussion of vitamins, we focus our attention on vitamin D since it can offer valuable adjunctive therapy for pregnancy if correctly administered [56] during the luteal phase (and not in the follicular phase) of the menstrual cycle.

A general comment must be made on the addition of vitamins belonging to the B complex. These vitamins are directed at reducing serum homocysteine levels, a well-known risk factor for cardiovascular diseases (CVD). One must consider that the normal content of folic acid (vitamin B9) found in many inositol PCOS supplements can prevent hyperhomocysteinemia. It may be deleterious to administer metformin to women with PCOS since metformin therapy has been associated with a significant reduction of vitamin B12 levels, particularly in overweight/obese and hyperinsulinemic patients [57]. Since the increase of homocysteine could be due to the decrease in its essential cofactors (folic acid and vitamin B12), we conclude that metformin may lead to hyperhomocysteinemia as an independent risk factor for CVD in patients with PCOS [57]. However, this is not the case with inositol administration in PCOS. Indeed, folic acid is sufficient, and the addition and the overload of B-complex vitamins become pharmacologically without meaning.

Although the inclusion of alpha-lactalbumin to MI has its therapeutic rationale, as shown before, the addition of alpha-lipoic acid to MI seems questionable since its presence is redundant in consideration of the composition of dietary supplements based on inositol. The normal daily dosage of 4 g MI plus DCI (40 : 1) can guarantee complete therapeutic coverage by means of a consistent and homogeneous formulation of inositols. Instead, in some products, the reduction of their dosage is balanced by the addition of alpha-lipoic acid [58, 59], used as an insulin sensitizer [60]. The therapeutic rationale for this combination, as well as its competitive advantage in the therapy of PCOS, seems to be totally lacking.

Finally, with regard to the product SelectSIEVE® containing phlorizin, chlorogenic acid, and quercetin, we remind the reader of the reduction of MI's intestinal absorption caused by these substances, as already previously highlighted [45].

## 8. Conclusions

Preclinical and clinical studies support the 40 : 1 MI/DCI ratio as the best one for PCOS treatment directed at restoring ovulation in these patients. Furthermore, it was demonstrated that DCI activity is beneficial mainly in a specific ratio with MI, whereas the progressive increase in concentration of DCI causes the parallel loss of the beneficial effects at the reproductive level with concomitant blastocyst quality. Furthermore, DCI negatively interferes with MI absorption at the intestinal level. Therefore, the dietary supplementation of MI may require being modulated based on the combined formulation used; the association of MI with DCI or with inhibitors of sugar's intestinal absorption and/or sugars may require higher dosage of MI to match the reference dosage of MI alone, particularly when the objective is to achieve a specific MI plasma level. Last, but certainly not least, we cannot overlook the pivotal finding that D-chiro-inositol is an aromatase inhibitor with resultant increased androgens and thus harmful consequences in infertile (and especially already hyperandrogenic PCOS) woman. In consideration of this, it is quite surprising to see that available proposed treatments for PCOS also include high dosages of DCI and often seem to be chosen without consideration of the therapeutic rationale and target. Overall, apart from a very few cases, the enrichment of inositols, used for PCOS, with other bioactive molecules has proved to be excessive and redundant, devoid of any scientific meaning.

## Figures and Tables

**Table 1:** List of different MI/DCI ratios used in the dietary supplements administered to PCOS patients.

MI/DCI ratio	
1	0.4 : 1
2	0.8 : 1
3	3.6 : 1
4	5 : 1
5	7 : 1
6	8 : 1
7	9.5 : 1
8	10 : 1
9	19.4 : 1
10	**40 : 1**
11	66 : 1
12	92 : 1
13	104 : 1

**Table 2:** Substances added to inositol in some dietary supplements for PCOS. They may be present alone or in combination with others on this list.

*Macroelements and microelements with relative dosages*
Magnesium (80 mg)
Chromium (50 *μ*g)
Iron (14 mg)
Manganese (2.5, 5, and 10 mg)
Copper (750 *μ*g)
Selenium (41.5 and 100 *μ*g)
Zinc (7.5, 11.25, and 15 mg)
Methylsulfonylmethane (100 mg) as source of sulfur
*Vitamins and precursors*
Beta-carotene (3 mg)
Nicotinamide, the amide form of vitamin B3 or niacin (60 mg)
Vitamin B6 (15 mg)
Vitamin B12 (1.5 *μ*g)
Vitamin C (80 mg)
Vitamin D (5, 12.5, and 50 *μ*g)
Vitamin E (12 mg)
*Other molecules*
Alpha-lactalbumin (50 mg)
Alpha-lipoic acid (500 mg)
Glucomannan (2 g)
L-Arginine (30 mg)
L-Glutathione (12 mg)
N-Acetyl cysteine (NAC) (150 mg)
Para-aminobenzoic acid (12.5 mg)
Revifast 80 mg (resveratrol and magnesium hydroxide)
MI without DCI is combined also with the product SelectSIEVE® (composition: phlorizin, chlorogenic acid, and quercetin)

## References

[1] Wong Y.-H. H., Kalmbach S. J., Hartman B. K., Sherman W. R. (1987). Immunohistochemical staining and enzyme activity measurements show myo-inositol-1-phosphate synthase to be localized in the vasculature of brain. *Journal of Neurochemistry*.

[2] Lewin L. M., Yannai Y., Melmed S., Weiss M. (1982). myo-inositol in the reproductive tract of the female rat. *International Journal of Biochemistry*.

[3] Sun T.-H., Heimark D. B., Nguygen T., Nadler J. L., Larner J. (2002). Both myo-inositol to chiro-inositol epimerase activities and chiro-inositol to myo-inositol ratios are decreased in tissues of GK type 2 diabetic rats compared to Wistar controls. *Biochemical and Biophysical Research Communications*.

[4] Heimark D., McAllister J., Larner J. (2014). Decreased myo-inositol to chiro-inositol (M/C) ratios and increased M/C epimerase activity in PCOS theca cells demonstrate increased insulin sensitivity compared to controls. *Endocrine Journal*.

[5] Monastra G., Unfer V., Harrath A. H., Bizzarri M. (2017). Combining treatment with myo-inositol andD-chiro-inositol (40:1) is effective in restoring ovary function and metabolic balance in PCOS patients. *Gynecological Endocrinology*.

[6] Facchinetti F., Dante G., Dante I. (2016). The ratio of MI to DCI and its impact in the treatment of polycystic ovary syndrome: experimental and literature evidences. *ISGE Series*.

[7] Unfer V., Carlomagno G., Papaleo E., Vailati S., Candiani M., Baillargeon J.-P. (2014). Hyperinsulinemia alters myoinositol to d-chiroinositol ratio in the follicular fluid of patients with PCOS. *Reproductive Sciences*.

[8] Bevilacqua A., Bizzarri M. (2016). Physiological role and clinical utility of inositols in polycystic ovary syndrome. *Best Practice & Research Clinical Obstetrics & Gynaecology*.

[9] Croze M. L., Soulage C. O. (2013). Potential role and therapeutic interests of myo-inositol in metabolic diseases. *Biochimie*.

[10] Croze M. L., Vella R. E., Pillon N. J. (2013). Chronic treatment with myo-inositol reduces white adipose tissue accretion and improves insulin sensitivity in female mice. *The Journal of Nutritional Biochemistry*.

[11] Larner J., Huang L. C., Tang G. (1988). Insulin mediators: structure and formation. *Cold Spring Harbor Symposia on Quantitative Biology*.

[12] Cheang K. I., Baillargeon J.-P., Essah P. A. (2008). Insulin-stimulated release of D-chiro-inositol-containing inositolphosphoglycan mediator correlates with insulin sensitivity in women with polycystic ovary syndrome. *Metabolism*.

[13] Baillargeon J.-P., Diamanti-Kandarakis E., Ostlund R. E., Apridonidze T., Iuorno M. J., Nestler J. E. (2006). Altered D-chiro-inositol urinary clearance in women with polycystic ovary syndrome. *Diabetes Care*.

[14] Nestler J. E., Jakubowicz D. J., Reamer P., Gunn R. D., Allan G. (1999). Ovulatory and metabolic effects of D-chiro-inositol in the polycystic ovary syndrome. *New England Journal of Medicine*.

[15] Iuorno M. J., Jakubowicz D. J., Baillargeon J.-P. (2002). Effects of d-chiro-inositol in lean women with the polycystic ovary syndrome. *Endocrine Practice*.

[16] Chiu T. T. Y., Tam P. P. L. (1992). A correlation of the outcome of clinical in vitro fertilization with the inositol content and embryotrophic properties of human serum. *Journal of Assisted Reproduction and Genetics*.

[17] Chiu T. T. Y., Rogers M. S., Law E. L. K., Briton-Jones C. M., Cheung L. P., Haines C. J. (2002). Follicular fluid and serum concentrations of myo-inositol in patients undergoing IVF: relationship with oocyte quality. *Human Reproduction*.

[18] Papaleo E., Unfer V., Baillargeon J.-P. (2007). Myo-inositol in patients with polycystic ovary syndrome: a novel method for ovulation induction. *Gynecological Endocrinology*.

[19] Gerli S., Papaleo E., Ferrari A., Di Renzo G. C. (2007). Randomized, double blind placebo-controlled trial: effects of myo-inositol on ovarian function and metabolic factors in women with PCOS. *European Review for Medical and Pharmacological Sciences*.

[20] Genazzani A. D., Lanzoni C., Ricchieri F., Jasonni V. M. (2008). Myo-inositol administration positively affects hyperinsulinemia and hormonal parameters in overweight patients with polycystic ovary syndrome. *Gynecological Endocrinology*.

[21] Zacchè M. M., Caputo L., Filippis S., Zacchè G., Dindelli M., Ferrari A. (2009). Efficacy of myo-inositol in the treatment of cutaneous disorders in young women with polycystic ovary syndrome. *Gynecological Endocrinology*.

[22] Costantino D., Minozzi G., Minozzi E., Guaraldi C. (2009). Metabolic and hormonal effects of myo-inositol in women with polycystic ovary syndrome: a double-blind trial. *European Review for Medical and Pharmacological Sciences*.

[23] Laganà A. S., Vitagliano A., Noventa M., Ambrosini G., D’Anna R. (2018). Myo-inositol supplementation reduces the amount of gonadotropins and length of ovarian stimulation in women undergoing IVF: a systematic review and meta-analysis of randomized controlled trials. *Archives of Gynecology and Obstetrics*.

[24] Unfer V., Carlomagno G, Rizzo P, Raffone E, Roseff S (2011). Myo-inositol rather than D-chiro-inositol is able to improve oocyte quality in intracytoplasmic sperm injection cycles. A prospective, controlled, randomized trial. *European Review for Medical and Pharmacological Sciences*.

[25] Carlomagno G., Unfer V., Roseff S. (2011). The D-chiro-inositol paradox in the ovary. *Fertility and Sterility*.

[26] Unfer V., Facchinetti F., Orrù B., Giordani B., Nestler J. (2017). Myo-inositol effects in women with PCOS: a meta-analysis of randomized controlled trials. *Endocrine Connections*.

[27] Artini P. G., Di Berardino O. M., Papini F. (2013). Endocrine and clinical effects of myo-inositol administration in polycystic ovary syndrome. A randomized study. *Gynecological Endocrinology*.

[28] Pizzo A., Laganà A. S., Barbaro L. (2014). Comparison between effects of myo-inositol andd-chiro-inositol on ovarian function and metabolic factors in women with PCOS. *Gynecological Endocrinology*.

[29] Nordio M., Proietti E. (2012). The combined therapy with myo-inositol and D-chiro-inositol reduces the risk of metabolic disease in PCOS overweight patients compared to myo-inositol supplementation alone. *European Review for Medical and Pharmacological Sciences*.

[30] Benelli E., Ghianda S. D., Cosmo C. D., Tonacchera M. (2016). A combined therapy with myo-inositol and D-chiro-inositol improves endocrine parameters and insulin resistance in PCOS young overweight women. *Int J Endocrinol*.

[31] Pkhaladze L., Barbakadze L., Kvashilava N. (2016). Myo-inositol in the treatment of teenagers affected by PCOS. *International Journal of Endocrinology*.

[32] Emekçi Özay Ö., Özay A. C., Çağlıyan E., Okyay R. E., Gülekli B. (2017). Myo-inositol administration positively effects ovulation induction and intrauterine insemination in patients with polycystic ovary syndrome: a prospective, controlled, randomized trial. *Gynecological Endocrinology*.

[33] Zeng L., Yang K. (2018). Effectiveness of myoinositol for polycystic ovary syndrome: a systematic review and meta-analysis. *Endocrine*.

[34] Bevilacqua A., Dragotto J., Giuliani A., Bizzarri M. (2019). Myo-inositol and D-chiro-inositol (40:1) reverse histological and functional features of polycystic ovary syndrome in a mouse model. *Journal of Cellular Physiology*.

[35] Gilling-Smith C., Willis D. S., Beard R. W., Franks S. (1994). Hypersecretion of androstenedione by isolated thecal cells from polycystic ovaries. *The Journal of Clinical Endocrinology & Metabolism*.

[36] Nordio M., Basciani S., Camajani E. (2019). The 40:1 myo-inositol/D-chiro-inositol plasma ratio is able to restore ovulation in PCOS patients: comparison with other ratios. *European Review for Medical and Pharmacological Sciences*.

[37] Ravanos K., Monastra G., Pavlidou T., Goudakou M., Prapas N. (2017). Can high levels of D-chiro-inositol in follicular fluid exert detrimental effects on blastocyst quality?. *European Review for Medical and Pharmacological Sciences*.

[38] Nestler J. E., Jakubowicz D. J., Falcon de Vargas A., Brik C., Quintero N., Medina F. (1998). Insulin stimulates testosterone biosynthesis by human thecal cells from women with polycystic ovary syndrome by activating its own receptor and using inositolglycan mediators as the signal transduction System1. *The Journal of Clinical Endocrinology & Metabolism*.

[39] Harwood K., Vuguin P., DiMartino-Nardi J. (2007). Current approaches to the diagnosis and treatment of polycystic ovarian syndrome in youth. *Hormone Research in Paediatrics*.

[40] Longcope C. (1986). 1 Adrenal and gonadal androgen secretion in normal females. *Clinics in Endocrinology and Metabolism*.

[41] Buggs C., Rosenfield R. L. (2005). Polycystic ovary syndrome in adolescence. *Endocrinology and Metabolism Clinics of North America*.

[42] Edmunds S. E. J., Stubbs A. P., Santos A. A., Wilkinson M. L. (1990). Estrogen and androgen regulation of sex hormone binding globulin secretion by a human liver cell line. *The Journal of Steroid Biochemistry and Molecular Biology*.

[43] Penttilä T.-L., Koskinen P., Penttilä T.-A., Anttila L., Irjala K. (1999). Obesity regulates bioavailable testosterone levels in women with or without polycystic ovary syndrome. *Fertility and Sterility*.

[44] Sacchi S., Marinaro F., Tondelli D. (2016). Modulation of gonadotrophin induced steroidogenic enzymes in granulosa cells by d-chiroinositol. *Reproductive Biology and Endocrinology*.

[45] Garzon S., Lagana A. S., Monastra G. (2019). Risk of reduced intestinal absorption of myo-inositol caused by D-chiro-inositol or by glucose transporter inhibitors. *Expert Opinion on Drug Metabolism & Toxicology*.

[46] Schneider S. (2015). Inositol transport proteins. *FEBS Letters*.

[47] Aouameur R., Da Cal S., Bissonnette P., Coady M. J., Lapointe J.-Y. (2007). SMIT2 mediates all myo-inositol uptake in apical membranes of rat small intestine. *American Journal of Physiology-Gastrointestinal and Liver Physiology*.

[48] Leung K.-Y., Mills K., Burren K. A., Copp A. J., Greene N. D. E. (2011). Quantitative analysis of myo-inositol in urine, blood and nutritional supplements by high-performance liquid chromatography tandem mass spectrometry. *Journal of Chromatography B*.

[49] Ehrenkranz J. R. L., Lewis N. G., Ronald Kahn C., Roth J. (2005). Phlorizin: a review. *Diabetes/Metabolism Research and Reviews*.

[50] Monastra G., Sambuy Y., Ferruzza S., Ferrari D., Ranaldi G. (2018). Alpha-lactalbumin effect on myo-inositol intestinal absorption: in vivo and in vitro. *Current Drug Delivery*.

[51] Montanino Oliva M., Buonomo G., Calcagno M., Unfer V. (2018). Effects of myo-inositol plus alpha-lactalbumin in myo-inositol-resistant PCOS women. *Journal of Ovarian Research*.

[52] Mendoza N., Diaz-Ropero M. P., Aragon M. (2019). Comparison of the effect of two combinations of myo-inositol and D-chiro-inositol in women with polycystic ovary syndrome undergoing ICSI: a randomized controlled trial. *Gynecological Endocrinology*.

[53] Mendoza N., Galan M. I., Molina C. (2019). High dose of d-chiro-inositol improves oocyte quality in women with polycystic ovary syndrome undergoing ICSI: a randomized controlled trial. *Gynecological Endocrinology*.

[54] Brusco G. F., Mariani M. (2013). Inositol: effects on oocyte quality in patients undergoing ICSI. An open study. *European Review for Medical and Pharmacological Sciences*.

[55] Januszewski M., Issat T., Jakimiuk A. A., Santor-Zaczynska M., Jakimiuk A. J. (2019). Metabolic and hormonal effects of a combined Myo-inositol and d-chiro-inositol therapy on patients with polycystic ovary syndrome (PCOS). *Ginekologia Polska*.

[56] Monastra G., De Grazia S, De Luca L, Vittorio S, Unfer V (2018). Vitamin D: a steroid hormone with progesterone-like activity. *European Review for Medical and Pharmacological Sciences*.

[57] Esmaeilzadeh S., Gholinezhad-Chari M., Ghadimi R. (2017). The effect of metformin treatment on the serum levels of homocysteine, folic acid, and vitamin B12 in patients with polycystic ovary syndrome. *Journal of Human Reproductive Sciences*.

[58] Genazzani A. D., Prati A., Marchini F., Petrillo T., Napolitano A., Simoncini T. (2019). Differential insulin response to oral glucose tolerance test (OGTT) in overweight/obese polycystic ovary syndrome patients undergoing to myo-inositol (MYO), alpha lipoic acid (ALA), or combination of both. *Gynecological Endocrinology*.

[59] Fruzzetti F., Fidecicchi T., Palla G., Gambacciani M. (2020). Long-term treatment with alpha-lipoic acid and myo-inositol positively affects clinical and metabolic features of polycystic ovary syndrome. *Gynecological Endocrinology*.

[60] Estrada D. E., Ewart H. S., Tsakiridis T. (1996). Stimulation of glucose uptake by the natural coenzyme -lipoic acid/thioctic acid: participation of elements of the insulin signaling pathway. *Diabetes*.

